# Comparison of sequential CT arterioportography-arteriosplenography with standard cross-sectional imaging and endoscopy in children with portal hypertension

**DOI:** 10.1038/s41598-022-10454-y

**Published:** 2022-04-21

**Authors:** Simone Hammer, Florian Zeman, Hans Jürgen Schlitt, Christian Stroszczynski, Barbara Greiner, Michael Christian Doppler, Wibke Uller

**Affiliations:** 1 Department of Radiology, University Hospital Regensburg, Franz-Josef-Strauss-Allee 11, 93053 Regensburg, Germany; 2 Center for Clinical Trials, University Hospital Regensburg, Franz-Josef-Strauß-Allee 11, 93053 Regensburg, Germany; 3 Department of Surgery, University Hospital Regensburg, Franz-Josef-Strauss-Allee 11, 93053 Regensburg, Germany; 4 Department of Diagnostic and Interventional Radiology, Medical Center-University of Freiburg, Faculty of Medicine, University of Freiburg, Hugstetter Straße 55, 79106 Freiburg, Germany

**Keywords:** Medical imaging, Tomography

## Abstract

In this study the diagnostic capability and additional value of sequential CT arterioportography–arteriosplenography (CT AP–AS) in comparison to standard cross-sectional imaging and upper gastrointestinal endoscopy (UGE) in pediatric portal hypertension (PH) was analyzed. Patients with clinical signs of PH who underwent CT AP–AS in combination with additional contrast-enhanced magnetic resonance imaging (CE-MR) and/or contrast-enhanced computed tomography (CE-CT) were included. Two radiologists reviewed independently imaging regarding the capability to prove patency of (1) extrahepatic and intrahepatic main stem portal vein (PV), (2) intrahepatic PV system and (3) splenomesenteric venous axis. Imaging was reviewed for detection of abdominal varices and results were compared to UGE. Main venous supply of varices (PV and/or splenic vein system) and splenorenal shunting were evaluated. 47 imaging studies (20 CT AP-AS, 16 CE-MR, 11 CE-CT) and 12 UGE records of 20 patients were analyzed. CT AP–AS detected significantly more splenorenal shunts (*p* = 0.008) and allowed more confident characterization of the extra-/intrahepatic PV-system and splenomesenteric veins in comparison to CE-MR (*p* < 0.001). Extra- and intrahepatic PV-system were significantly more confidently assessed in CT AP–AS than in CE-CT (*p* = 0.008 and < 0.001 respectively). CT AP–AS was the only modality that detected supply of varices and additional gastric/duodenal varices. In this retrospective study CT AP–AS was superior to standard cross-sectional imaging concerning confident assessment of the venous portosplenomesenteric axis in pediatric patients. CT AP–AS detected additional varices, splenorenal shunting and supply of varices.

## Introduction

Biliary atresia and extrahepatic portal vein obstruction (EHPVO) with development of cavernous transformation are the most common causes of PH in children^1–3^. The impaired hepatopetal blood flow from the susperior mesenteric vein (SMV) and splenic vein (SV) leads to congestion of the portomesenteric venous system with development of collateral pathways resulting in varices and portosystemic shunts. Splenomegaly and upper gastrointestinal variceal hemorrhage are the most common initial clinical manifestations of pediatric PH^4,5^. Management consists of preventing and controlling variceal hemorrhage by pharmacological and endoscopic treatment. Radiologic-interventional (transjugular intrahepatic portosystemic shunting) and surgical techniques (portosystemic shunting, liver transplantation) are alternative therapy options in case of endoscopic or pharmacologic treatment failure^6^.

In patients with recurrent gastrointestinal bleeding despite medical and endoscopic therapy^7^, cross-sectional imaging is essential for assessing the whole portomesenteric venous system as well as the SV for choosing adequate therapeutic management. The extent of PV thrombosis to the splanchnic veins and intrahepatic PV as well as patency of the SV, SMV, recessus of Rex and the intrahepatic branches of the PV are crucial to decide on the best therapy options^1^.

Sequential CT AP–AS is based on sequential contrast application into the splenic artery (SA) and the superior mesenteric artery (SMA) during CT and proved to evaluate individual venous hemodynamic changes in children with symptomatic PH^8^. This technique enables maximal contrast filling of the targeted abdominal veins, differentiation of the main supply of varices and determination of blood flow direction.

The aim of this study was to assess the diagnostic capability of CT AP–AS regarding the patency of the portal and splenomesenteric veins in comparison to standard cross-sectional imaging (CE-CT and CE-MR). Moreover the present study was designed to analyze delineation of varices in CT AP–AS in comparison to standard cross-sectional imaging and upper gastrointestinal endoscopy (UGE).

## Results

### Study cohort

**Figure 1:**
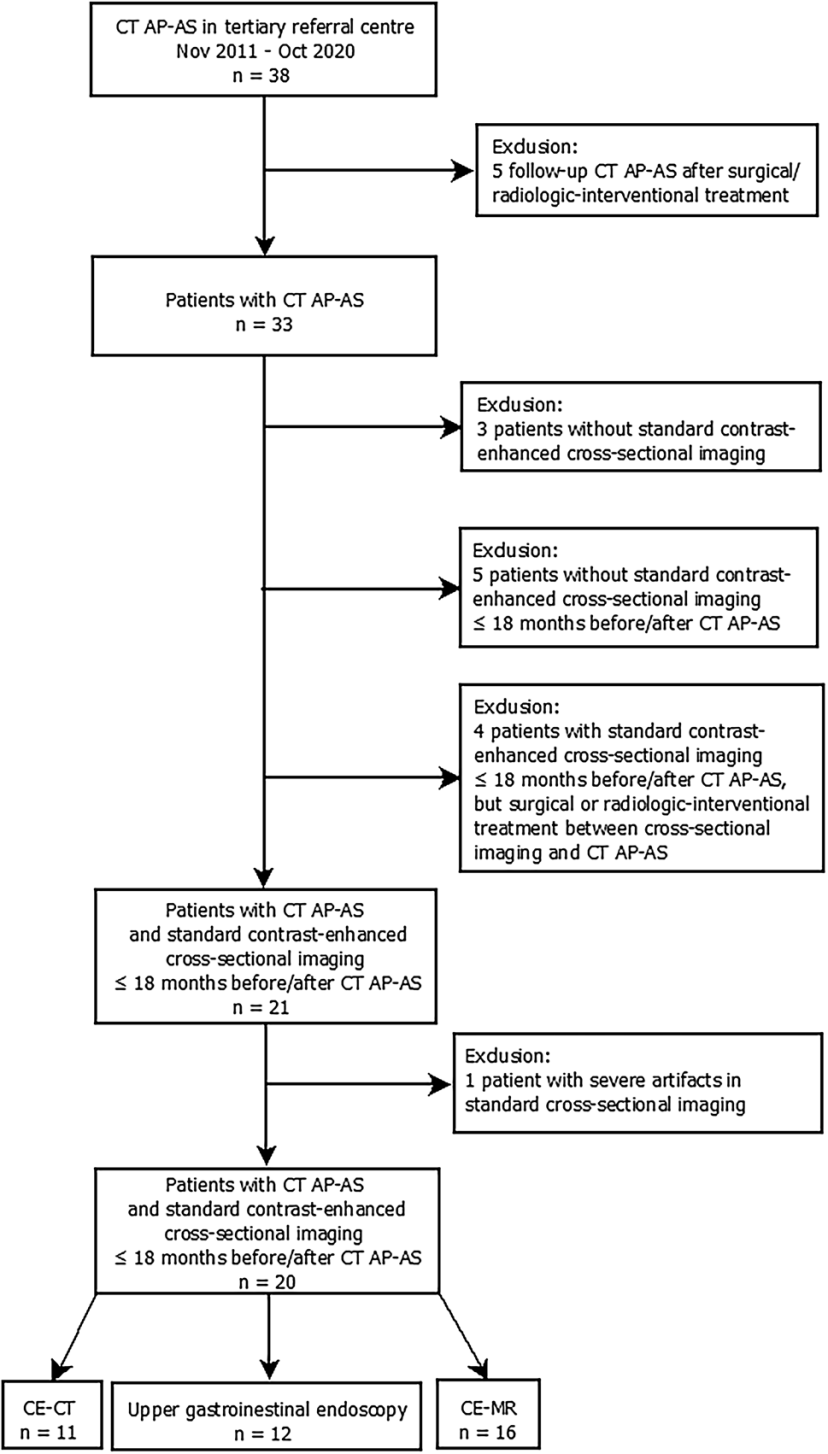
Flowchart of the study cohort.

Between November 2011 and October 2020 38 CT AP–AS were conducted in 33 pediatric patients (flowchart of the study cohort, Fig. 1). 5 follow-up CT AP–AS were excluded. For 8 patients standard cross-sectional imaging was not available or standard-cross sectional imaging was outside the defined time-interval (18 months before or after CT AP-AS). Another 4 patients were excluded as they underwent surgical or radiological-interventional treatment between standard cross-sectional imaging and CT AP-AS. Severe artifacts in standard cross-sectional imaging led to exclusion of 1 patient. Cause of PH in the 13 excluded patients (*n* = 6 female; median age 8.5 years, range 2.5–15.7 years) was prehepatic in 10 cases and intrahepatic in 3 cases. 20 CT AP–AS performed in 20 patients were included in this study (*n* = 9 female; median age 13.6 years, range 1.7–18.4 years; median body weight 37.2 kg; range 14.1–80.6 kg). Etiology of PH was prehepatic in 15 cases and intrahepatic in 5 cases. 11 patients underwent CE-CT and 16 patients CE-MR (7 patients underwent both, CE-CT and CE-MR). Median time interval between CT AP–AS and standard cross-sectional imaging was −2.4 months (range −16.3–14.8 months). Medical records of UGE were available in 12 patients. Median time interval between CT AP–AS and UEG was −0.4 months (range −3.9–7.1 months).

### Comparison CT AP–AS and standard cross-sectional imaging

#### Evaluation portosplenomesenteric veins

Results for diagnostic capability are shown in Table 1. CT AP–AS allowed confident characterization of the extra- and intrahepatic main stem PV (region 1) in all cases. Status of extrahepatic and intrahepatic main stem PV was significantly better characterized with CT AP–AS compared to CE-CT (*p* = 0.008) and CE-MR (*p* < 0.001).

**Table 1:** Diagnostic capability CT AP–AS versus CE-CT and CT AP–AS versus CR-MR (frequency of confident characterization per imaging modality and vessel).

Vessel	CT AP-AS	CE-CT	*p* values	CT AP-AS	CE-MR	*p* values
	(*n* = 11)			(*n* = 16)		
Region 1	33/33 (100)	24/33 (72.7)	**0.008**	48/48 (100)	33/48 (68.8)	** < 0.001**
Extrahepatic PV	11/11 (100)	8/11 (72.7)	0.24	16/16 (100)	12/16 (75.0)	0.13
Main stem intrahepatic PV	11/11 (100)	6/11 (54.5)	0.07	16/16 (100)	11/16 (68.8)	0.07
Cavernous transformation main stem PV	11/11 (100)	10/11(90.9)	1.00	16/16 (100)	10/16 (62.5)	**0.041**
Region 2	52/53 (98.1)	25/53 (48.4)	** < 0.001**	72/72 (100)	41/72 (56.9)	** < 0.001**
Left central intrahepatic PV^a^	11/11 (100)	5/11 (45.5)	**0.041**	16/16 (100)	9/16 (56.3)	**0.023**
Left peripheral intrahepatic PV^b^	11/11 (100)	7/11 (63.6)	0.13	16/16 (100)	9/16 (56.3)	**0.023**
Right central intrahepatic PV^a, c^	10/10 (100)	5/10 (50.0)	0.07	12/12 (100)	7/12 (58.3)	0.07
Right peripheral intrahepatic PV^b, c^	9/10 (90.0)	2/10 (20.0)	**0.023**	12/12 (100)	5/12 (41.7)	**0.023**
Cavernous transformation intrahepatic PV	11/11 (100)	6/11 (54.5)	0.07	16/16 (100)	10/16 (62.5)	**0.041**
Region 3	32/33 (97.0)	26/33 (78.8)	0.08	45/48 (93.8)	31/48 (64.6)	**0.001**
SMV	11/11 (100)	9/11 (81.8)	0.48	15/16 (93.8)	11/16 (68.8)	0.22
SV	11/11 (100)	9/11 (81.8)	0.48	16/16 (100)	13/16 (81.3)	0.25
SMV/SV confluence	10/11 (90.9)	8/11 (72.7)	0.63	14/16 (87.5)	7/16 (43.8)	**0.023**

Values are number of studies, numbers in parentheses are percentages. *p* values are written in bold when statistically significant.

^a^contrast enhancement within the right/left PV to first branching.

^b^contrast enhancement within the expected course of the first order intrahepatic left/rigth PV extending peripherally.

^c^*n* = 1 and *n* = 4 patients after split-liver transplantation in the CE-CT group and CE-MR group respectively.

**Figure 2:**
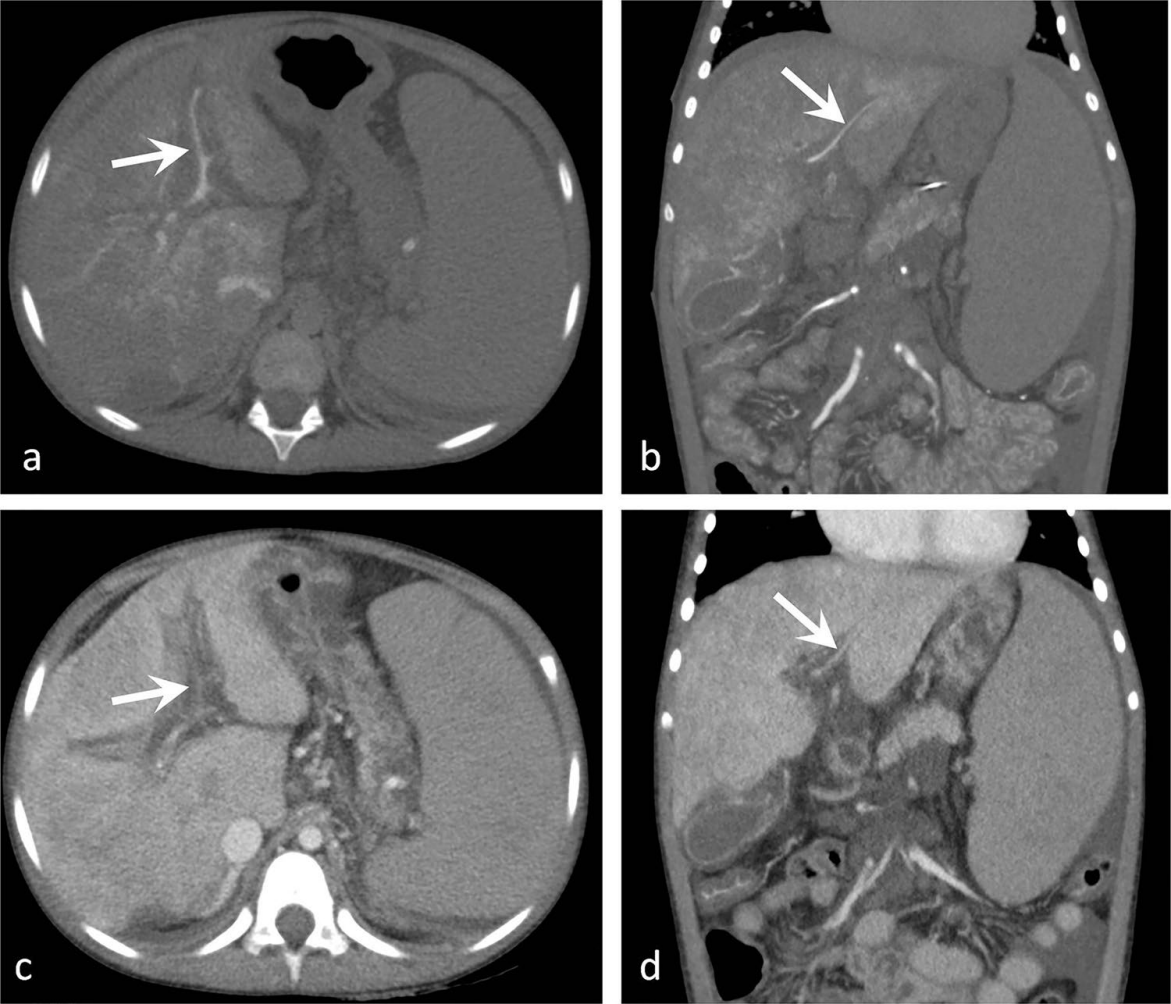
A 8-year-old female patient with PH. Coronal and axial images of CT-AP (**a**, **b**) and CE-CT (**c**, **d**). The left central and peripheral intrahepatic PV (arrows) were categorized "not reliably assessable" in CE-CT (**c**, **d**). CT-AP delineates the small, but clearly patent left intrahepatic PV.

**Figure 3:**
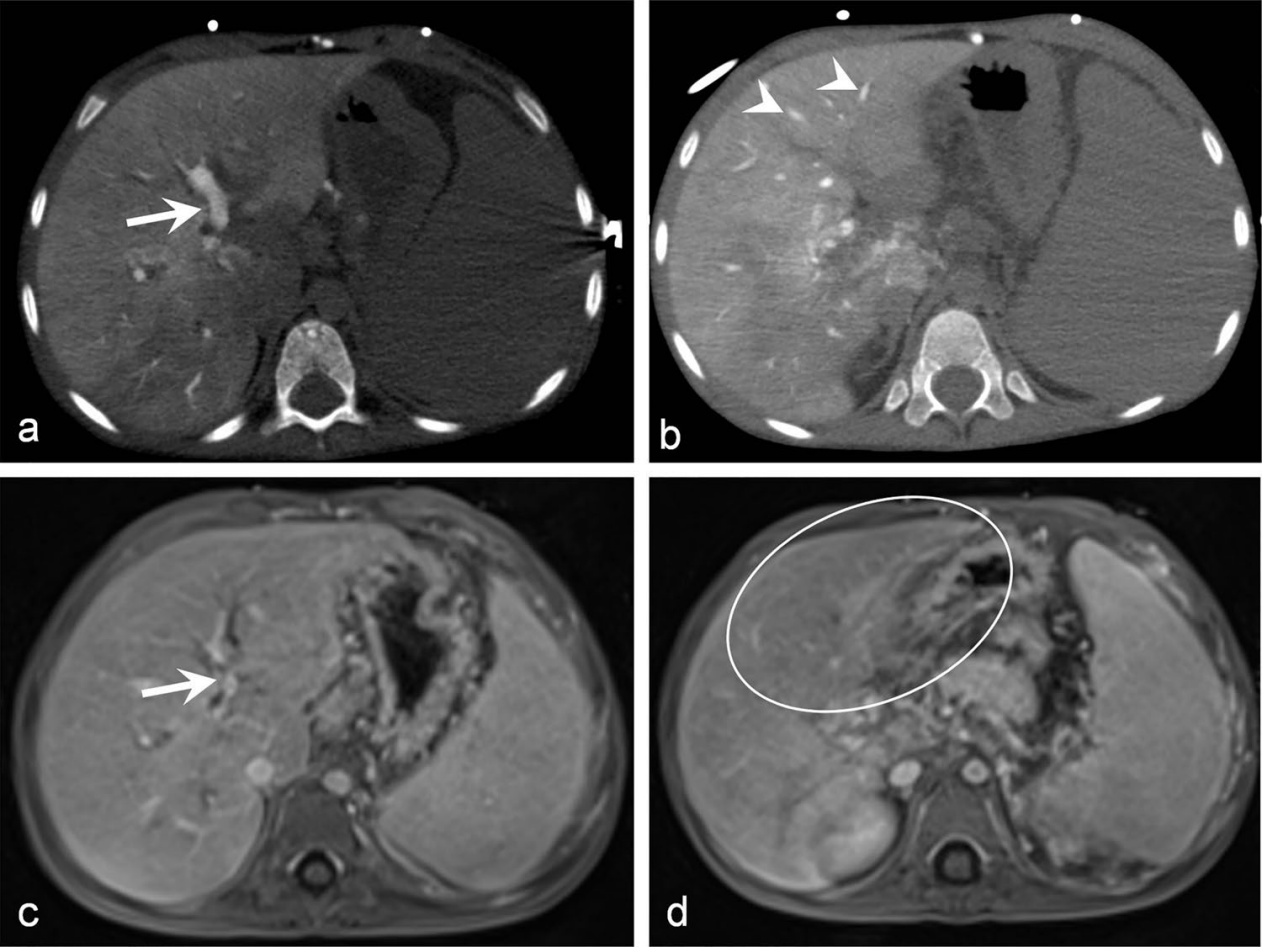
A 6-year-old male patient with EHPVO. Axial images of CT-AP (**a**, **b**) and CE-MR (**c**, **d**). The left central (arrows) and peripheral PV (arrowheads) were not confidently assessed in CE-MR, especially for the peripheral left PV CE-MR was non-diagnostic (circle in **d**).

The intrahepatic PV system (region 2) was confidently assessed in CT AP-AS, only in 1 patient the right peripheral intraheptic PV was assigned to the category "not reliably assessable" (52/53, 98.1%). Confident categorization in CE-CT and CE-MR for the intrahepatic PV system (region 2) was possible for 25/53 intrahepatic portal veins (48.4%) and 41/72 intrahepatic portal veins (56.9%) respectively (*p* < 0.001 and < 0.001). Ability to determine status of the left central intrahepatic PV was significantly better at CT AP–AS compared to CE-CT (*p* = 0.041) and CE-MR (*p* = 0.023). Capability to diagnose status of the right central intrahepatic vein increased with use of CT AS-AP compared to CE-CT (*p* = 0.07) and CE-MR (*p* = 0.07). For determination of the left peripheral branches differences between CT AS-AP and CE-MR were significant (*p* = 0.023). Concerning the right peripheral PV, CT AS-AP provided the highest diagnostic capability (*p* = 0.023) in comparison with CE- CT and CE-MR (Figs. 2 and 3).

Differences between CT AP–AS and CE-MR were significant concerning the diagnostic capability of extra- and intrahepatic cavernous transformation (*p* = 0.041). Cavernous transformation of the PV was equally diagnosed at CE-CT and CT AP–AS with a trend towards higher diagnostic capability at CT AP–AS concerning intrahepatic cavernous transformation (*p* = 0.07).

CT AP–AS allowed confident assessment of the splenomesenteric veins (region 3) for 32/33 (97%) vessels in the CT AP–AS vs. CE-CT group and for 45/48 (93.8%) vessels in the CT AP–AS vs. CE-MR group. For region 3 confident diagnosis at CE-CT was possible for 26/33 (78.8%) veins and at CE-MR for 31/48 (64.6%) veins (*p* = 0.08 and 0.001 respectively).

With respect to the SMV and SV CT AP-AS, CE-CT and CE-MR performed equally. Concerning the confluence, CT AP–AS and CE-CT also performed equally whereas CE-MR differed significantly from CT AP–AS results (*p* = 0.63 and 0.023 respectively).

#### Evaluation varices and splenorenal shunting

CT AP–AS delineated significantly more varices compared to CE-CT: stomach (*p* = 0.041), duodenum (*p* = 0.041) and small intestinum (*p* = 0.023).

**Figure 4:**
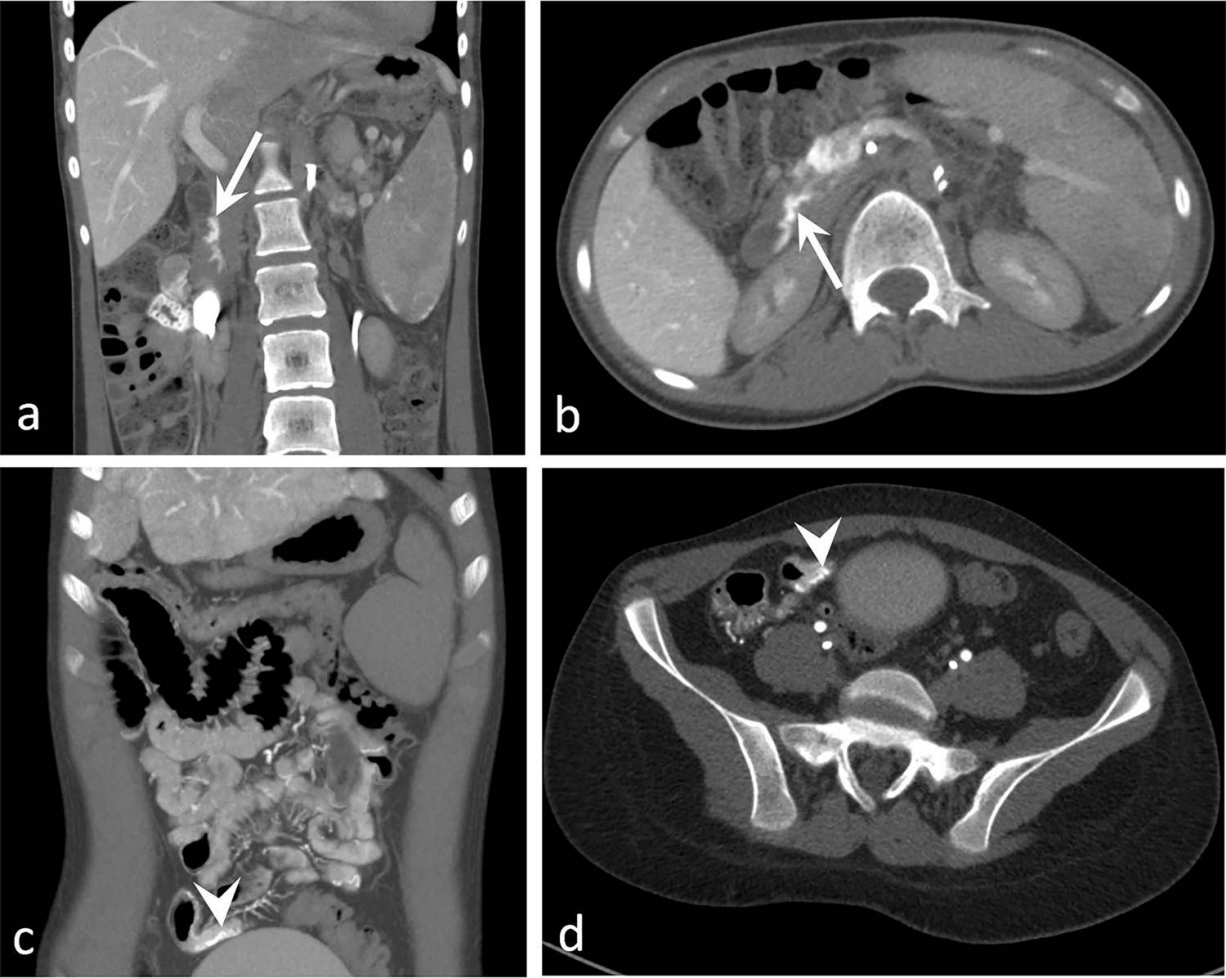
Axial and coronal images of CT-AP of two 16-year-old male patients with congenital hepatic fibrosis (**a**, **b**) and hepatoportal sclerosis (**c**, **d**). CT-AP detected small duodenal varices not described in UEG (arrow) and small intestinal varices (arrowheads) not detectable in CE-CT.

In comparison to CE-MR CT AP–AS shows significantly more gastric (*p* = 0.002), duodenal (*p* = 0.013), small intestinal (*p* = 0.004) and colonic (*p* = 0.041) varices. There was no significant difference between CT AP–AS and standard cross-sectional imaging for esophageal/paraesophageal varices. Figure 4 illustrates small intestinal varices only detectable using CT AP-AS.

CT AP–AS delineated main venous supply of varices (SMV versus SV system): esophagus (2/15 SMV, 8/15 SV, 5/15 both), paraesophageal space (3/14 SMV, 7/14 SV, 4/14 both), stomach (12/18 SV, 6/18 both), duodenum (9/10 SMV, 1/10 both), small intestinum (13/13 SMV), colon (9/9 SMV), rectum (3/8 SMV, 4/8 SV, 1/8 both).

Significantly more spontaneous splenorenal shunts were detected in CT AP–AS compared to CE-MR (*p* = 0.008).

### Comparison CT AP–AS and UEG

**Figure 5:**
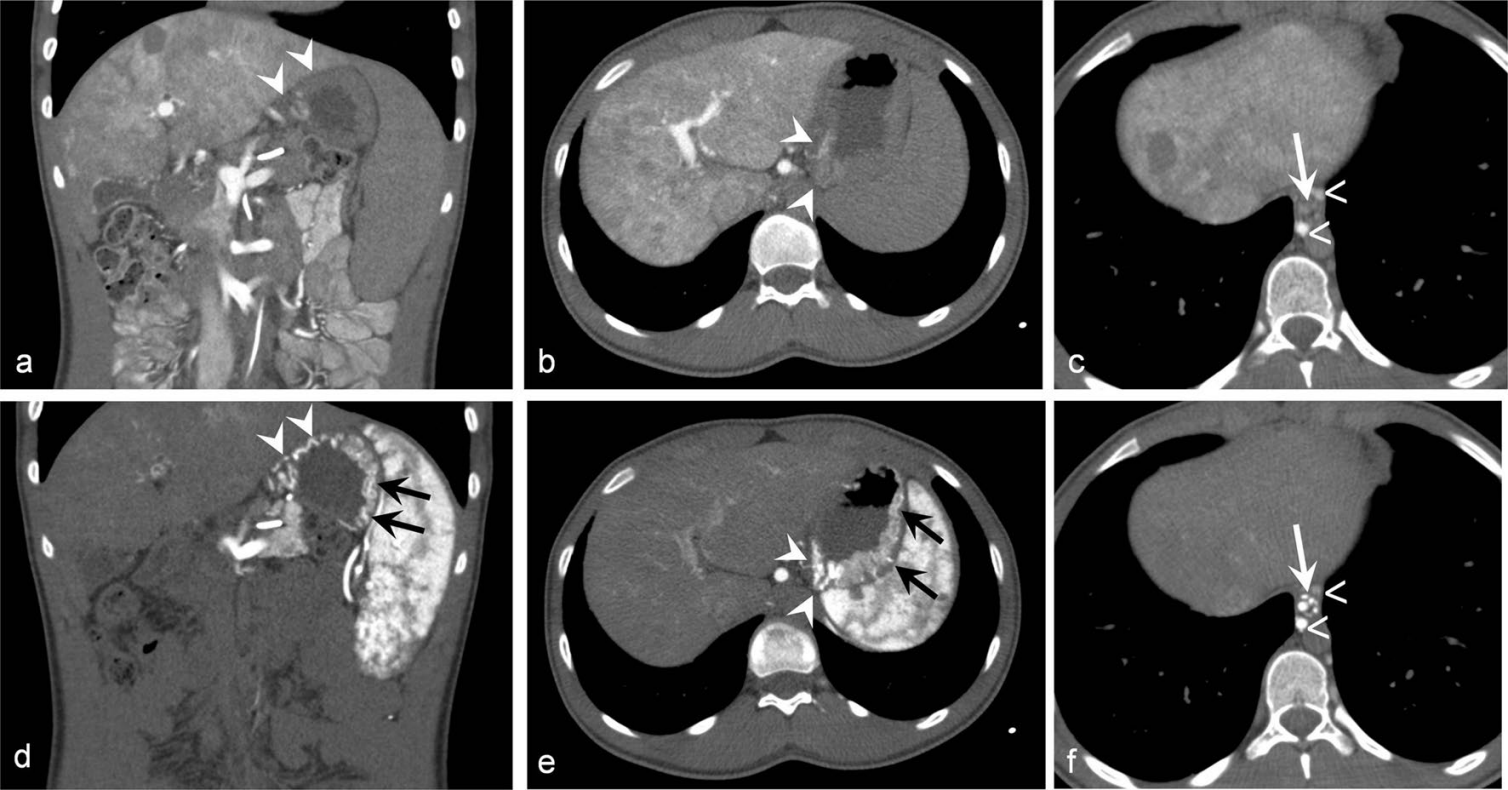
A 15-year-old male patient with PH. Coronal and axial images of CT-AP (**a**–**c**) and CT-AS (**d**–**f**). Enhancement of varices of the gastric cardia (arrowheads) and esophagus (white arrows) predmominates in CT-AS (**d**–**f**) in comparison to CT-AP (**a**–**c**). Enhancement of paraesophageal varices (open arrows) in both CT-AP (**c**) and CT-AS (**f**). In UEG varices at the gastric cardia were described, CT-AS (**d**, **e**) delineates additional small fundic varices (black arrows).

CT AP–AS identified 11/12 endoscopic described esophageal, 12/12 gastric and 2/2 duodenal varices. In 6/12 cases CT AP–AS showed additional varices in the fundus and corpus of the stomach (Fig. 5). Furthermore CT AP–AS detected in 2 patients small duodenal varices not described in UGE (Fig. 4).

## Discussion

In comparison to standard cross-sectional imaging CT AP–AS allowed significantly more confident assessment of the extra- and intrahepatic PV system. Especially the left central and peripheral intrahepatic PV were more confidently characterized in CT AP–AS compared to CE-MR. In contrast to CE-CT CT AP–AS facilitates significantly more confident assessment of the intrahepatic portalvenous system as a whole (98.1% for CT AP–AS versus 48.4% for CE-CT) and in particular of the left central PV (100% for CT AP–AS versus 45.5% for CE-CT). The low performance of CE-CT regarding the intrahepatic portalvenous system is remarkable as, in our experience, CE-CT is very reliable for the evaluation of the portalvenous system in the adult population. In pediatric patients, however the contrast-to-noise ratio (most probable due to insufficient contrast-medium timing) is often not sufficient for confident diagnosis. Furthermore, perfect timing in pediatric patients is very individual and volume of contrast is restricted to the body weight of the child. Our results regarding the assessment of the intrahepatic portalvenous system meet the findings of Kwan et al. They compared the diagnostic capability of different invasive/non-invasive imaging modalities (CE-CT, CE-MR, ultrasound, transsplenic portography, transhepatic portography, transarterial portography and CT-AP) for preoperative meso-Rex bypass planning in pediatric patients. CT-AP was available for only 6 cases, but interestingly, in contrast to all other imaging modalities CT-AP allowed confident assessment of the crucial structures for the meso-Rex bypass procedure (left intrahepatic PV, PV contiguity and SMV) in all cases. All but one of these patients (5/6) underwent non-diagnostic imaging studies prior to CT-AP. Therefore Kwan et al. concluded, that CT-AP should be the first or second line imaging modality for preoperative planning. They proposed CE-CT for first assessment, because CE-CT in portalvenous phase depicts also the SV and renal veins. This is important when key criteria for the meso-Rex bypass procedure are not fulfilled and distal splenorenal shunting is an alternative. If CE-CT is not conclusive, CT-AP should be performed^9^. Shneider et al.^6^ proposed transjugular retrograde or percutaneous transhepatic portal venography, if CE-CT or CE-MR are non-diagnostic for the intrahepatic PV system. However, this invasive technique lacks the possibility for postprocessing to generate 2D and 3D reconstructions and determination of the exact size of intrahepatic portal vein branches^10^^.^ Parekh et al.^11^ showed that 4D flow MR imaging allows 3D visualization of the PV and quantification of PV hemodynamics in a small pediatric study cohort. However, since PV system was not assessed in detail and results were not compared to any other imaging modalities, the value of this technique in the workup of pediatric PH remains unclear.

CT AP–AS delineated significantly more varices of the stomach, duodenum and small intestinum compared to CE-CT and CT-MR despite standard cross-sectional imaging is suitable to depict upper gastrointestinal varices in adults and children with PH^12–14^. CT AP–AS displays excellent venous contrast due to the direct contrast injection into the SA and the SMA. In contrast to peripheral intravenous contrast injection dilution effects do not occur and accordingly, higher detection rate of varices results. The excellent delineation of abdominal varices and moreover, direction of flow is essential for surgical treatment planning.

In contrast to standard cross-sectional imaging CT AP–AS revealed small varices in more uncommon locations like the duodenum, small intestinum and colon. Varices in these locations are of interest because they can be origin of occult gastrointestinal hemorrhage. Though systematic data regarding these varices are scarce and restricted to endoscopy. A recent study based on esophageal capsule endosopy conducted for screening and surveillance purposes showed a prevalence of 0.07% for duodenal varices^15^. Kumar et al.^16^ found colonic varices in 3.7% of colonoscopies in pediatric patients with EHPVO. The prevalence of duodenal and colonic varices using CT AP–AS in this series was much more higher (10/20 and 9/20 patients respectively). CT AP–AS detected endoscopic described esophageal varices in all but one case but additional gastric varices in 6/12 cases and additional duodenal varices in 2/12 cases. Gulati et al. compared CE-CT imaging of the esophageal and gastric vasculature in pediatric EHPVO with UGE. In their study, CE-CT showed a false positivity of 20% for gastric varices compared to UEG. Interestingly, endoscopy detected varices in all false positive cases during follow up. Since endoscopic evaluation is restricted to the inner surface of the gastric wall, veins within the gastric wall or smaller varices may be missed due to air insufflations during endoscopy^17^. CE-CT and CT AP–AS probably pick up changes in the gastric vasculature earlier because this technique visualizes both the superficial and the intramural veins. The even higher percentage of false positive findings for gastric varices in our study may be explained by the higher venous contrast in CT AP–AS and the better image quality in comparison to the CE-CT imaging studies of Gulati et al.

Moreover, combination of CT-AP and CT-AS differentiates the main venous supply of varices (SMV versus SV system)^8^. To our knowledge no other image modality provides this hemodynamic information. According to the experience of our interdisciplinary team of interventional radiologists, surgeons and gastroenterologists main venous supply of varices is important for planning of successful therapeutic interventions in order to decompress the SMV or SV system. However further systematic evaluation of the relevance of these findings for the therapeutic outcome is necessary.

CT AP–AS detected significantly more spontaneous splenorenal shunts compared to CE-MR. Three-dimensional CE-MR is a standard imaging technique suitable for assessment of portosystemic collaterals including spontaneous splenorenal shunts in children^18,19^. CT imaging like CT AP–AS however has higher spatial resolution and is less prone to motion artifacts which may explain the higher detection rate of splenorenal shunts in CT AP-AS. Additionally due to the direct contrast medium injection into the SA the renal veins are not opacified in CT AP-AS, consequently even very small splenorenal shunts are detectable. Spontaneous splenorenal shunting is of utmost interest for interventional access routes and planning pediatric liver transplantation, as (large) splenorenal shunts can cause postoperative PV flow steal syndrome which may require ligation of the splenorenal shunt^20^. Recent studies evaluated the clinical relevance of spontaneous portosystemic shunts including splenorenal shunting on CT imaging in adult liver cirrhosis and found an association of spontaneous portosystemic shunts with higher mortality and higher complication rate for PV thrombosis, hepatic encephalopathy and gastrointestinal hemorrhage^21,22^. Equivalent studies for pediatric PH do not yet exist, but these findings underline the importance of a comprehensive imaging evaluation of patients with PH.

The major limitations of this study are its retrospective design and the small study cohort. There might be a selection bias because only complex patients underwent CT AS-AP and 13 patients were excluded owing to a lack of CE-CT or CE-MR (within the defined period). The defined time interval between standard cross-sectional imaging and CT AP–AS (18 months before or after CT AP-AS) was chosen arbitrarily and might seem rather long. Only complicated patients in need for surgical or radiologic-interventional treatment underwent CT AP–AS due to its invasive nature. In many cases CE-CT or CE-MR was done for initial diagnostics and CT AP–AS later in case of deterioration. As CT AP–AS delivers all therapy-relevant information, standard cross-sectional imaging was often not repeated. Patency of the portalvenous system and the development of portosystemic collaterals are dynamic, to overcome this bias CE-CT/CE-MR conducted before AND after CT AP–AS were analyzed. UGE was the only reference standard regarding upper gastrointestinal varices. Analysis of CT AP–AS concerning the patency of portosplenomesenteric veins was restricted to diagnostic capability because comprehensive reference standards like intraoperative findings do not exist in every patient.

In conclusion, CT AP–AS with direct contrast-application in the SA and SMA via arterial transfemormal catheterization is a novel, invasive technique that guides evaluation of complex anatomic and hemodynamic changes in complicated pediatric PH more confident in comparison to standard cross-sectional imaging and delineates variceal formation.

## Methods

This single center study was conducted according to the principles expressed in the Declaration of Helsinki. The ethics committee of the University of Regensburg approved this retrospective study (approval no. 20-2123-104) and waived the requirement for informed consent. Pediatric patients (18 years old or younger) with clinical signs of PH who underwent CT AP–AS at our tertiary referral university hospital center with available standard contrast-enhanced cross-sectional imaging (CE-CT in portalvenous phase and/or CE-MR with axial T1-weighted dynamic three-dimensional gradient-echo sequences) within 18 months before or after CT AP–AS were included in this study. Follow-up CT AP–AS and standard cross-sectional imaging studies with severe artifacts were excluded. Medical records of UGE were reviewed when available.

### Imaging

CT AP–AS was conducted following a prospectively determined protocol as described in detail before^8^:

1. 4-F sheaths and 4-F catheters were used to catheterize the SMA and SA selectively by transfemoral approaches. Subsequently the patient was transferred to the CT suite with the two catheters secured at the groin.
2. CT-AP (contrast injection through the catheter placed in the SMA) and CT-AS (contrast injection through the catheter placed in the SA) were conducted at a helical 256 (2 × 128)-slice dual source CT scanner (Somatom Flash scanner; Siemens, Forchheim, Germany) with automatic tube voltage selection (80 kV or 100 kV) and automatic tube current modulation. 0.4 mL nonionic contrast agent per kilogram bodyweight diluted with the same volume of NaCl was injected at a rate of 2.5 mL/s.

Portalvenous phase of contrast-enhanced CT and dynamic T1-weighted three-dimensional gradient-echo sequences of CE-MR after peripheral intravenous contrast injection were evaluated.

### Imaging analysis

Two radiologists (12 and 14 years of experience in the interpretation of vascular imaging studies) reviewed independently imaging using a dedicated PACS viewer (Syngo; Siemens, Forchheim, Germany) in a random order. Time interval between the readout sessions were at least 4 weeks. Readers used a structured template. They were blinded to clinical data and results of imaging.

### Evaluation portosplenomesenteric veins

Patency of the extrahepatic/intrahepatic PV system, SMV, SV and SMV/SV confluence was evaluated. These vessels were summarized in 3 vessel regions. Region 1: extrahepatic and intrahepatic main stem PV. Region 2: intrahepatic PV system with left/right central intrahepatic PV (defined as the section between PV main trunk bifurcation and first branching of the intrahepatic PV) and left/right peripheral intrahepatic PV branches (defined as evident contrast enhancement of PV branches in the peripheral liver parenchyma). Region 3: SMV, SV, SMV/SV confluence. Status of the vessels was classified: (1) patent, (2) occluded, (3) not reliably assessable. If the readers assigned the same classification-type (patent or occluded) the overall assessment of the vessel was defined as “confident characterization”. If two readers assigned different categories the overall assessment of the regarding vessel was "not reliably assessable".

### Evaluation varices and splenorenal shunting

Varices (paraesophageal space, esophagus, stomach, duodenum, small intestinum, colon, rectum) and splenorenal shunting were reviewed in consensus. Medical records of UGE were reviewed concerning presence and localization of varices (esophagus, cardia/fundus/corpus of the stomach, duodenum).

Enhancement patterns of CT-AP and CT-AS were compared in consensus to differentiate variceal origin and main supply (SV system or SMV system).

### Statistical analysis

Data are presented as absolute and relative frequencies. CT AP–AS was compared to standard cross-sectional imaging by using the McNemar test. A *p*-value < 0.05 was considered statistically significant. All analyses were performed using *R version 4.1.0* (The R Foundation for Statistical Computing).

## Data Availability

The data that support the findings of this study are available within the article.
